# Effect of Fortified Human Milk on the Growth Parameters of Babies With Very Low Birth Weight

**DOI:** 10.7759/cureus.22889

**Published:** 2022-03-06

**Authors:** Ali Amjad, Abdul Rehman, Afaq hussain, Waqas Shakir, Aashee Nadeem, Nazia fatima

**Affiliations:** 1 Department of Neonatology, The Children’s Hospital & The Institute of Child Health, Multan, PAK

**Keywords:** neonat, preterm, gestational age, low birth weight babies, growth parameters, human milk, fortification

## Abstract

Objective

The objective of this study is to assess the effect of fortified human milk on growth parameters of very low birth-weight babies.

Study place and duration

This randomized controlled trial took place at the neonatal intensive care unit (NICU), Children's Hospital, and Institute of Child Health in Multan from the 1st of January 2020 to the 1st of July 2021.

Material and methods

In group I, 25ml human milk was fortified with a 1g human milk fortifier (HMF) sachet (1g of HMF gives 4kcal added to 25ml of human milk). In group II, newborns were fed preterm formula (493 kcal/100 g where 0.8 g=4 kcal added to 25 ml of human milk) mixed with human milk. Infants were administered human milk + olive oil (0.4 mL = 4Kcal per 25ml human milk) in group III. Everyday weight gain, digestive intolerance (vomiting and/or abdominal distension), sepsis, hospital stay, electrolyte imbalance (derangement of serum sodium, potassium, chloride, and magnesium levels), albumin, and cholesterol/triglyceride levels were assessed. The data was analyzed through descriptive and inferential means using Pearson's chi-square tests and one-way analysis of variance (ANOVA).

Results

Results indicate that preterm formula infants gain higher weight compared to human fortifier infants and olive oil. Similarly, the difference was statistically significant (p=0.001). However, olive oil infants gained a lower head circumference compared to the other two groups, and the difference was statistically significant as well (p=0.000). Moreover, feeding intolerance and electrolyte imbalance were higher in olive oil infants, p=0.020 and p=0.024, respectively.

Conclusion

It can be concluded that the use of and preterm formula can prove beneficial in increasing the growth rate in terms of weight gain, length gain, and head circumference.

## Introduction

Meeting the nutritional needs of a preterm infant is a big challenge because of certain conditions such as a decrease in the body's reserves, accelerated metabolism, reduced capacity of adaptation in the presence of electrolyte imbalance, and increased risk for complications related to the immaturity of the digestive system [1, 2]. Provision of nutrients to the newborns has similar concerns to the concerns of the intra-uterine life at the same gestational age, i.e., increase in the rate of physical growth, etc. [3]. The most preferred method to provide nutrients to very low-weight newborns is via maternal milk, as it contains enough fats, proteins, calories, minerals, and electrolytes. Additionally, it helps in maintaining gastrointestinal and cognitive functions, specific bio-active functions, immunity against infections, and infant-mother bond development [4-6]. Even though there are numerous advantages of human milk, studies in the literature show that the growth of low-birth-weight newborns who were fed with human milk was exclusively slower than the growth during the intrauterine period [7]. Newborns with a weight of <1.5 kg at the time of birth and being given non-fortified maternal milk showed lower rates of growth with poor levels of serum phosphorus and calcium. Whereas low-weight newborns who were fed fortified maternal milk show an increased growth rate [8]. Thus, multiple studies have recommended the use of fortified maternal milk with the aim to meet the nutritional requirements [9-12] and to prevent bone demineralization in young babies [13].

A review of the literature has shown that very few studies could be identified in which two groups of low-birth-weight newborns were compared who were fed maternal milk exclusively during the study duration. It can be attributed to the fact that the collection of enough maternal milk in raw form is a very challenging process during the hospitalization period of low-birth-weight newborns. Moreover, fortifiers are of high cost. Thus, in this study, we are going to compare the weight gain, height gain, and clinical complications in the low-birth-weight newborns (weighing <1.5 kg) being fed fortified maternal milk, preterm formula added to human milk, and olive oil added maternal milk to the point that their weight reaches to 1.8 kg.

## Materials and methods

The study was conducted from the 1st of January 2020 to the 1st of July 2021 on 96 preterm newborn babies at a gestational age of ≤34 weeks and weighing <1500 g at birth admitted to the neonatal intensive care unit (NICU). In this randomized controlled study, newborns whose mothers were capable of expressing milk and who met the criteria of inclusion were divided into three groups randomly. The group that received pure human milk plus human milk fortifier was said to be group I, the one receiving preterm formula was said to be group II, and group III preterm babies were given human milk plus olive oil. During the period of study, out of 130 preterm neonates admitted in NICU with a weight of <1500 g, 29 were not included in the study because they were fed other milk types, four expired due to sepsis, and one of them had necrotizing enterocolitis before the beginning of the 100 mL/kg/day diet. Neurological impairment, congenital malformation, and impairment of the gastrointestinal tract were also added to the exclusion criteria. Sequenced numbering was used for each group for double-blind randomization by drawing lots carried out by the on-duty physicians. The researchers were blind to the groups of the newborns, and the outcomes of randomizations were documented. The milk was collected from the mothers who expressed milk in an exclusive and was stored in the separate milk storage room. Nurses in the storage room added a fortifier in the milk as per the protocols of the study, according to which intervention group the newborn was in, and the milk was given to the newborn in the NICU.

The study conducted by Martin et al. was used as a reference for calculating sample size [14]. Written consent was signed from the parents or guardians of the newborn. The Children’s Hospital and The Institute of Child Health Multan approved this study. At birth, parenteral nutrition of 60 kcal/kg/day was given to a newborn weighing less than 1500 g on the first day. For drainage of the gastric residue, an open orogastric tube was used. When the clinical status and diet acceptance were good, newborns were categorized according to gender and age and fed on fortified milk, preterm formula, and human milk with added olive oil. It is worth mentioning here that the method of increasing each milk was unified. In group I, a concentration of 1 g sachet of human fortifier milk was added to 25 ml of human milk (1 g of human milk fortifier (HMF) gives 4 kcal added to 25 ml of human milk). In group II, newborns were fed preterm formula (493 kcal/100 g where 0.8 g=4 kcal added to 25 ml of human milk) mixed with human milk. In group III, newborns were fed human milk plus olive oil (0.4 mL = 4Kcal added to 25ml of human milk). At the end of the period, the weight was compared after the use of a fortifier and gaining anticipated weight - the newborns were shifted to the intermediate care unit until they reached 2 kg and were breastfed by the mother, which is the criteria for discharge from the hospital.

Daily weight gain, feeding intolerance, i.e., vomiting and/or abdominal distension, weekly head circumference and length gain, sepsis, hospital stay, electrolyte imbalance (derangement of serum sodium, potassium, chloride, and magnesium levels), serum levels of albumin, cholesterol, and triglycerides were the variables that were assessed. The presence of symptoms clinically and positive blood and urine culture was used to define the sepsis episode. Model BP Baby and Filizola™, with an accuracy of 125 to 2,500 g, were the digital scales that were used for measuring the weight of newborns at the time of birth and daily. When the newborn was fed for the third time in the morning, the weight of the newborn was observed. If the gastric tube was being used, 5 g was subtracted from the total weight of the newborn, and 20 g was subtracted if the venous access device was used. Follow-ups were done until the newborn reached a weight of 1800 g. The length of the newborn and the circumference of the head were measured each week with the help of inextensible measuring tape.

The descriptive statistics of mean, standard deviation, and frequencies were demonstrated. Fisher's exact test or Pearson's chi-square test were used for analyzing the relation and comparing the variables between groups. The Student's t-test was used for parametric variables, and the Mann-Whitney test was used for non-parametric variables. A value of p<0.05 was considered significant for the association.

## Results

Ninety-six infants were included in this study. Briefly, 32 (33.3%) infants were given human milk fortifier (group I), 32 (33.3%) preterm formula (group II), and 32 (33.3%) were given olive oil (group III). 43.8% of preterm formula infants were small for gestational age (SGA), 28.1% of olive oil infants were SGA, while only (15.6%) human milk fortifiers were SGA, the difference was statistically significant (p=0.046). The mean baseline weight of olive oil infants was higher than the other two groups - the difference was statistically significant (p=0.015). While the mean baseline length of preterm was higher than the other two groups, the difference was statistically significant (p=0.045, Table 1).

**Table 1:** Baseline characteristics of the study groups AGA= appropriate for gestational age, SGA= small for gestational age *Chi-square test, † One way analysis of variance (ANOVA)

Characteristic	Human milk fortifier (group I) n=32 (33.3%)	Preterm formula (group II) n=32 (33.3%)	Olive oil (group III) n=32 (33.3%)	p-value
Gender				
Male	n=20 (62.5%)	n=17 (53.1%)	n=26(81.3%)	0.055*
Female	n=12 (37.5%)	n=15 (46.9%)	n=6(18.8%)	
SGA	n=5 (15.6%)	n=14 (43.8%)	n=9(28.1%)	0.046*
AGA	n=18 (56.3%)	n=19 (59.4%)	n=17(53.1%)	0.881*
Baseline Weight (g)	1228.28±43.29	1227.62±35.54	1229.41±45.26	0.015^†^
Baseline Length (cm)	34.94±3.59	36.11±3.32	33.93±3.41	0.045^†^

Preterm formula infants gained higher weight than the human fortifier and olive oil infants - the difference was statistically significant (p=0.001). Olive oil infants gain low head circumference compared to the other two groups, and the difference was statistically significant as well (p=0.000). Feeding intolerance and electrolyte imbalance was higher in olive oil infants, p=0.020 and p=0.024, respectively (Table 2).

**Table 2:** Comparison (mean ± standard deviation) of fortifiers on growth parameters *Chi-square test, † One way analysis of variance (ANOVA)

Characteristic	Human milk fortifier (group I) n=32 (33.3%)	Preterm formula (group II) n=32 (33.3%)	Olive oil (group III) n=32 (33.3%)	p-value
Weight gain (g/day)	25.64±1.92	26.56±2.21	23.61±2.71	0.001^†^
Length gain (cm/week)	1.04±0.73	1.20±0.99	0.76±0.94	0.138^†^
Head circumference gain (cm/week)	1.41±0.72	1.20±1.0	0.55±0.88	0.000^†^
Length of hospital stay (days)	30.80±2.26	30.74±2.03	31.02±2.04	0.855^†^
Sepsis	n=3 (9.4%)	n=3 (9.4%)	n=4 (12.5%)	0.894*
Feeding intolerance	n=2 (6.3%)	n=4 (12.5%)	n=10 (31.3%)	0.020*
Electrolyte imbalance	n=1 (3.1%)	n=3 (9.4%)	n=8 (25.0%)	0.024*
Serum albumin	4.37±1.17	3.88±1.11	3.52±1.38	0.023^†^
Serum cholesterol	165.41±45.22	156.59±28.18	174.11±22.84	0.118^†^
Serum triglyceride	130.46±45.68	140.83±27.16	132.44±42.66	0.536^†^

## Discussion

Weight gain in the newborns in the fortified maternal milk group was 25.64±1.92 g/day, while weight gain in newborns of preterm formula added to the maternal milk group was 26.56±2.21 g/d, and in a group of olive oil added to maternal milk, it was 23.61±2.71 g/d (p=0.001). Height gain was 1.20±0.99 cm in preterm formula added to the human milk group, while it was 1.04±0.73 cm in the group of newborns who were fed fortified human milk and 0.76±0.94 in group III (0.138). Multiple previous studies have shown that the use of fortified human milk is associated with increased weight gain and height gain in low-birth-weight newborns and also helps in the prevention of metabolic bone diseases [15-18]. Nonetheless, most past studies studied the role of formula milk from milk banks as a nutritional replacement to raw human milk [19].

A systemic review comprising 13 studies was conducted on the role of fortifiers in human milk in providing nutrition to low-birth-weight newborns and to assess bone metabolism, physical growth, and neurological development [20]. The result of the systemic review suggested that fortified human milk can result in a short-term surge in growth, i.e., weight and height gain and an increase in head circumference. However, the long-term advantages of fortified human milk on neurological growth could not be assessed in that systemic review.

In a similar prospective randomized trial, it was seen that weight gain was 15.1 g/day and 12.9 g/day in groups with fortified human milk and non-fortified raw human milk feeding. Linear growth in the fortified human milk group was significantly higher (1.04 cm/week) than the pure human milk (0.86 cm/week) [21]. However, only 80% of the milk used in this past study was pure human milk; thus, their results cannot be compared to the current study. Similar results could also be seen in some other past studies [22, 23].

Clinical complications were similar in both groups with no statistically significant difference. However, previous studies have shown that the use of fortifiers was associated with an increased risk of necrotizing enterocolitis in low-birth-weight newborns because of increased osmolality [24]. This increase in osmolality of fortified human milk has been recognized as a risk factor for the development of necrotizing enterocolitis [25]. Nonetheless, the risk of developing necrotizing enterocolitis with the use of fortified human milk is still lower than formula milk [26, 27].

Limitations and future suggestions

The current study has a few limitations. Firstly, the study's findings are not generalizable due to the study's small sample size and diverse target group. Second, the randomization of the study's comparative effectiveness design is also a limitation. Thirdly, there were different formulas and HMF preparations, but the protein and calorie content were the same.

In the future, the importance of mother milk in the neurodevelopment and cognitive function of low-birth-weight infants will need to be reevaluated in light of current HMF and preterm formula compositions.

## Conclusions

Based on the above observations, it can be concluded that the use of human milk supplemented with preterm formula is superior to the other two types of milk in increasing the growth rate in terms of weight gain, length gain, and head circumference. Thus, it is a recommended way of feeding infants born prematurely, and multi-nutrient fortification can be safely initiated when the milk volume reaches 50-80 ml/kg/d. Overall, the efficacy, safety, and ethical implications of human milk-based fortifiers were investigated, as well as the benefit in terms of morbidity and mortality when infants were fed an exclusively human milk-based diet incorporating these products. 
